# Survivor Expectations and Experiences of One‐Stop Crisis Centres in Bangladesh: A Qualitative Study of Health System Responsiveness to Gender‐Based Violence

**DOI:** 10.1111/hex.70643

**Published:** 2026-03-27

**Authors:** Mst Sabrina Moonajilin, Sayema Mubashshira, Md Khalid Ibne Kamal, Rajib Ul Islam, Labina Taher, Tabeen Taher, Sabkat Kamal

**Affiliations:** ^1^ Department of Public Health and Informatics Jahangirnagar University Savar Dhaka Bangladesh; ^2^ Queensland University of Technology Brisbane Australia; ^3^ Department of Government & Politics Jahangirnagar University Savar Dhaka Bangladesh; ^4^ Kelvin Grove State College Brisbane Australia; ^5^ Department of Urban & Regional Planning Jahangirnagar University Savar Dhaka Bangladesh

**Keywords:** gender‐based violence, gender‐responsive public services, One‐Stop Crisis Centre

## Abstract

**Background:**

Gender‐based violence (GBV) constitutes a significant public health concern that necessitates coordinated, survivor‐centred health system responses. Health systems are essential in addressing GBV by delivering timely, respectful, and coordinated support to survivors. In Bangladesh, One‐Stop Crisis Centres (OCCs) have been established within public hospitals to provide integrated medical, psychosocial, and legal services. However, there is limited research on how survivors evaluate these services and whether their expectations for survivor‐centred care are fulfilled in practice. This study explored the perspectives of survivors and frontline providers regarding OCC services, with a specific focus on health system responsiveness.

**Methods:**

This qualitative study explored the experiences and expectations of survivors and service providers regarding OCC services in Bangladesh. In‐depth interviews were conducted with GBV survivors (*n* = 32) and frontline service providers (*n* = 4) at two sites. Data were analysed using reflexive thematic analysis, guided by a health system responsiveness framework lens and the gender‐responsive public services framework (availability, accessibility, acceptability, quality, and accountability) to explore how institutional practices shape survivor‐centred care.

**Results:**

Five interrelated themes emerged. Survivors demonstrated limited awareness of OCC services before crisis events, with access frequently facilitated by police or emergency referrals. Institutional capacity constraints, such as staffing shortages and inadequate infrastructure, negatively impacted the timeliness of care. Experiences related to dignity and privacy were inconsistent. Some survivors reported supportive interactions, whereas others described judgmental questioning and breaches of confidentiality. Fragmented coordination among medical, legal, and social services disrupted the continuity of care. The absence of formal feedback mechanisms restricted opportunities for survivors to contribute to service improvement.

**Conclusions:**

Although OCCs constitute a significant institutional response to GBV care and the realities of service delivery. Enhancing awareness pathways, improving intersectoral coordination, and establishing survivor‐centred accountability mechanisms could improve the responsiveness of integrated GBV services.

**Acronyms**
COREQ: consolidated criteria for reporting qualitative research
GBV: gender‐based violence
GRPS: gender‐responsive public services
IDI: in‐depth interview
LMIC: low‐ and middle‐income country
OCC: One‐Stop Crisis Centre
WHO: World Health Organization

## Patient or Public Contribution

Survivors were not directly involved in the study design due to ethical and safety concerns related to engaging individuals with a lived experience of GBV during the planning stage. However, the research design prioritised survivor perspectives by placing survivor narratives at the centre of data collection and analysis. Survivors' accounts directly informed the identification of key service challenges and guided the interpretation of findings related to health system responsiveness. During interviews, participants were invited to reflect on potential improvements to OCC services. The study team intends to disseminate findings through stakeholder engagement with policymakers, service providers, and organisations supporting survivors of GBV, ensuring that survivor perspectives inform future service improvement initiatives.

## 1 Introduction

Gender‐based violence (GBV) constitutes a pervasive public health issue with substantial physical, psychological, and social consequences for survivors. Health systems play a critical role in responding to GBV by providing immediate medical care, psychosocial support, and facilitating access to legal and social services [1]. Recent discourse has shifted focus from the mere availability of services to the responsiveness of health systems, defined as the extent to which services align with user expectations concerning dignity, access, confidentiality, and continuity of care [2, 3, 4]. GBV does more than cause immediate physical harm. It leads to long‐term health issues like psychological trauma, social isolation, and reduced access to education and work [5]. Patient expectations are central to assessing health system responsiveness. These expectations represent the standards of care individuals anticipate when engaging with health services [6]. Expectations are influenced by prior experiences, prevailing social norms, and the level of institutional trust. Failure to meet these expectations can diminish service utilisation, trust, and user engagement [7, 8]. In many low‐ and middle‐income countries (LMICs), structural barriers, including fragmented service systems, limited institutional resources, and pervasive social stigma, often constrain health systems' capacity to fulfil these expectations [9, 10, 11].

Numerous countries have implemented integrated service delivery models to support GBV survivors. One‐Stop Crisis Centres (OCCs) are intended to provide coordinated medical, legal, and psychosocial services at a single site, to reduce secondary victimisation and enhance access to care [12, 13]. Initially developed in high‐income countries and subsequently adapted in LMICs, OCCs are generally situated within public hospitals and function at the intersection of health, justice, and social welfare sectors [14, 15]. Evidence from various contexts indicates that OCCs can enhance survivor experiences when they are sufficiently resourced, effectively coordinated, and tailored to local needs. Nevertheless, ongoing challenges include staff shortages, fragmented coordination, and limited survivor engagement [12, 16].

In Bangladesh, OCCs were established in public hospitals as a flagship initiative to deliver integrated, survivor‐centred services for women experiencing violence. These centres aim to reduce barriers to care by consolidating medical treatment, counselling, legal aid, and referrals within a single location [14]. Despite national policies that emphasise comprehensive support, limited evidence exists regarding how OCCs are experienced by users or whether these services meet survivors' expectations. While prior studies have investigated service utilisation and administrative structures, limited research has examined survivors' evaluations of these services and the extent to which their expectations of care are met in practice [17]. Since the early 2000s, OCCs established in public hospitals nationwide have played a vital role in providing integrated support and contributing to national efforts to address and reduce violence against women. Previous research on OCCs in Bangladesh has primarily examined service structures, utilisation patterns, and administrative challenges, with limited attention to survivors' perspectives [14, 18]. This study addresses this gap by investigating how survivors and frontline providers perceive OCC services, with particular emphasis on access, quality of care, coordination, and opportunities for survivor input within the health system

Existing literature predominantly emphasises institutional design or policy objectives, with comparatively little focus on the lived experiences of survivors and frontline staff [19]. Examining lived experiences is especially significant in the South Asian context, where social stigma, patriarchal norms, and geographic disparities influence survivors' access to public services [20]. Survivors residing in rural or semi‐urban areas, as well as younger and unmarried women, frequently encounter additional obstacles related to mobility, confidentiality, and fear of community repercussions [21, 22]. Analysing OCC operations within these social and institutional contexts necessitates consideration of not only service availability but also accessibility, acceptability, quality, and accountability from both user and provider perspectives.

This study applies the gender‐responsive public services (GRPS) framework to analyse how health system structures influence survivor experiences and expectations of care, in accordance with calls for increased responsiveness and accountability in health services. The framework conceptualises public services through five interconnected dimensions: Service readiness & continuity, equitable access to care, dignity, respect, and trust, survivor‐perceived quality and responsiveness [23]. In this research, GRPS is utilised to organise and interpret survivor and provider narratives, rather than to conduct a formal evaluation of service performance [24, 25]. Despite the expansion of hospital‐based GBV services across LMICs, evidence remains limited concerning survivor experiences with integrated care and their expectations in South Asia [26]. Most evaluations focus on service availability or utilisation, with less attention given to responsiveness dimensions that are important to service users, including dignity, confidentiality, prompt attention, and continuity [23]. In Bangladesh, social stigma and fragmentation between legal and medical systems discourage care‐seeking. Understanding survivor experiences is essential for strengthening OCCs beyond their formal establishment [27]. This study explored how survivors and frontline providers describe the accessibility, acceptability, quality, coordination, and accountability of OCC services, and identified gaps between expectations and service delivery. By examining survivor narratives within these dimensions, the study aims to elucidate how institutional arrangements influence survivors' experiences of care and to identify gaps between expected and actual responsiveness.

## 2 Methods

This study used a qualitative research design to explore survivor experiences and expectations of care in Dhaka and Cox's Bazar. In‐depth interviews (IDIs) with GBV survivors, service providers, and institutional stakeholders assessed how well OCCs meet survivors' needs and the quality of services. IDIs explore participants' thoughts and motivations in depth and are especially valuable for sensitive topics. They also allow researchers to adapt questions and explore emerging themes during the conversation [28]. Expectations were examined retrospectively by analysing participants' accounts regarding the provision of safety, dignity, confidentiality, and coordinated support by crisis services, as well as the alignment of these expectations with their actual experiences.

### 2.1 Participants

Data were collected from two OCCs located in Dhaka and Cox's Bazar, representing urban and semi‐urban service contexts, respectively. The study was conducted at two OCCs in Dhaka and Cox's Bazar. Dhaka, the capital city, contains Bangladesh's largest tertiary hospitals and primarily serves an urban population. In contrast, Cox's Bazar, located approximately 390 km southeast of Dhaka, is a coastal district with higher poverty rates, limited health infrastructure, and a substantial displaced population. Selecting these two sites enabled examination of OCC experiences across distinct socio‐economic and service‐delivery contexts. These sites were purposively selected due to their operational maturity and geographic contrast, enabling comparative insights into service delivery across socio‐political landscapes. Participants were recruited using purposive and snowball sampling methods, ensuring a diversity of perspectives while prioritising ethical sensitivity and trust‐building. The determination of sample size was based on the principles of information power and thematic saturation. Recruitment and interviews proceeded until consecutive sessions yielded no substantively new codes or service‐process issues, as determined through a saturation grid and analytic memos. The survivor sample (*n* = 32) was selected to ensure variation by site, relationship to perpetrator, and service pathway, thereby supporting saturation within the study's focused aims. Additionally, OCC staff members (*n* = 4) were case managers and counsellors and were included to triangulate survivor accounts with frontline service perspectives regarding coordination, constraints, and accountability practices. Eligibility criteria included being at least 18 years old and having accessed OCC services within the previous 6 months. Participation was voluntary, and refusal did not affect the provision of services. OCC staff participants were recruited directly from the centres and included one case manager and one counsellor at each site. At the time of data collection, each OCC employed approximately staff members responsible for medical coordination, psychosocial support, and legal referrals.

### 2.2 Data Collection Tool

An interview guide was developed based on the GRPS framework to highlight the influence of gendered power relations on access to and experiences of public services. To ensure alignment with focus on service user experience, the GRPS pillars (availability, accessibility, acceptability, quality, accountability) were explicitly mapped to health system responsiveness domains such as prompt attention, dignity, confidentiality, and coordination or continuity. The guide examined survivors' expectations and experiences regarding OCC service delivery and gathered providers' perspectives on institutional capacity, referral pathways, and intersectoral coordination. The guide was available in Bengali and was applied flexibly to address emergent issues raised by participants (Supplementary file).

### 2.3 Data Collection

IDIs were conducted in Bangla and lasted 55‐90 min. A pilot test involving (*n* = 2) was undertaken to refine interview language and reduce the risk of stigma or reinforcement of negative stereotypes. Each interview commenced with rapport‐building questions and was followed by an overview of the study process. Following informed consent, participants took part in audio‐recorded, semi‐structured interviews. Safety protocols were implemented to protect both researchers and participants. Participants were informed of their right to skip questions, terminate the interview at any time, and access designated support contacts. Interviews were conducted by a female public health researcher with prior qualitative experience on sensitive topics, under the supervision and with debriefing support of the senior author during fieldwork. All interviews were audio‐recorded, and additional handwritten field notes were taken to capture nonverbal cues and contextual information. Field notes were taken to capture contextual information and the researcher's reflexive observations.

### 2.4 Data Analysis

The audio recordings of the IDIs were transcribed in Bengali first. The researcher read and re‐listened to the transcripts to become acquainted with the data and translated the Bengali version of the transcript into English. The researcher followed the forward‐back translation process [29]. Another native speaker of Bangladesh translated four randomly selected sample transcripts from the English language to the Bengali version. The Bengali versions of the transcripts were then compared with the original Bengali recordings for accuracy and consistency. Minor changes were made to the wording, but no changes were made to the data's meaning. Transcripts were anonymised and organised using NVivo (version 2020 R1.7.1). Initial open coding was conducted by the first author, who developed a provisional codebook that integrated inductive codes with deductive codes aligned to the GRPS pillars and responsiveness domains. Two co‐authors independently reviewed the codebook and a purposive subset of transcripts, representing both sites and participant groups, to assess coherence and critically examine interpretations. Discrepancies were addressed through discussion, and an audit trail documenting codebook iterations and analytic memos was maintained. Themes were refined using reflexive thematic analysis in accordance with Braun and Clarke's six‐step approach, with attention to patterned meaning, narrative divergence, institutional context, and gendered power relations [30]. Participant quotes are included to support transparency, while reflexive journaling and peer debriefing were employed to enhance credibility.

### 2.5 Researcher Positionality

The study's focus on health system responsiveness and institutional accountability was informed by the first author's background in public health and previous research on gender and health. Given the sensitive context of GBV and the hospital‐based setting, the research team anticipated power imbalances related to social class, education, and participants' reliance on services. To mitigate these risks, recruitment occurred after immediate crisis care was completed, participation was emphasised as voluntary, and interviews were conducted in a private space separate from routine service delivery. During interviews, the researcher employed non‐judgmental prompts, regularly assessed participant comfort, and refrained from seeking identifying details that could increase risk. Reflexivity was operationalised through contemporaneous field notes and a reflexive journal that documented assumptions, emotional responses, and emergent interpretations. These reflections were discussed during regular debriefings with co‐authors to examine how the researcher's positionality could influence rapport, questioning, and coding decisions. Analytic memos were employed to actively identify disconfirming cases, such as accounts of respectful care alongside reports of mistreatment, and to distinguish participant meanings from researcher interpretations. Transcripts were not returned to participants due to safety and confidentiality concerns in this setting; instead, credibility was supported through iterative member‐checking within interviews (summarising key points for confirmation) and triangulation with staff perspectives.

## 3 Results

The study sample included survivors of GBV (*n* = 32) and staff members from OCCs (*n* = 4). Survivors were recruited from OCCs located in Dhaka (*n* = 20) and Cox's Bazar (*n* = 12). Survivor participants were aged 18 years and older, with ages spanning from 18 to over 30 years. All survivor participants identified as women. Most had completed some level of formal education, although few had attained education beyond the secondary level.

Participants reported diverse marital statuses, including unmarried, married, and divorced. Several divorced participants reported leaving their marriages due to experiences of violence or concerns regarding personal safety. To protect privacy and mitigate stigma, participants disclosed only district‐level information about their residence. The OCC staff participants included case managers (*n* = 2) and counsellors (*n* = 2), with one individual in each role recruited from each site. The analysis revealed five themes that illustrate discrepancies between survivors' expectations for responsive care and the institutional realities of service delivery.

### 3.1 Theme 1: Unmet Expectations of Access

Participants consistently showed that survivors had limited awareness of OCCs. Most participants learned about these services only after a crisis, typically through referrals from police or medical staff. Reliance on intermediaries demonstrates how institutional gatekeeping limits survivors' access to care. Survivors from rural and semi‐urban areas faced additional barriers, such as long travel times and limited local information. These findings underscore the need for improved community outreach and reduced dependence on police referrals, which are not always accessible or safe for all survivors.

I had never heard of such a place before that day. No one in my village knew it existed. If the police officer hadn't insisted, I would have gone back home, thinking there was no help for someone like me. People talk about hospitals and courts, but never about this centre.

(Survivor, 9)

Unless the police bring them, many survivors don't even reach us. The system unintentionally makes the police a gatekeeper. This discourages women who are already afraid of law enforcement.

(OCC staff, Dhaka)

It took me more than 3 h to travel here, changing buses and walking long stretches. By the time I arrived, I was exhausted and frightened. Many women from my area wouldn't even attempt the journey.

(Survivor, 7)

### 3.2 Theme 2: Expectations Vs System Capacity

Participants reported significant shortages of trained personnel, especially during evenings, holidays, and emergencies. Both survivors and providers noted delays in service delivery and a lack of trauma‐informed care. These staffing gaps undermine comprehensive and timely crisis support. As a result, survivors faced long wait times and inconsistent access to psychosocial counselling. These challenges contribute to the perception that OCCs are not adequately equipped to address urgent needs.

When I came, the counsellor was not available. They told me to wait until the next day. I felt like my pain had to be put on hold. In that moment, I thought: if there is no one to listen now, then what is the meaning of a crisis centre?

(Survivor, 5)

We don't have enough staff to cover all shifts. If one person is absent, everything slows down. Survivors may have to wait hours just to give their statement or get medical care.

(OCC staff, Cox's Bazar)

Some staff are kind, but others don't know how to talk to survivors. Instead of comforting me, they kept repeating the same questions, like they didn't understand that answering them was painful. It felt like I was being interrogated, not helped.

(Survivor, 2)

### 3.3 Theme 3: Trust, Dignity, and Care Quality

Participants' accounts varied in this regard. Some described empathetic staff, while others reported judgement, invasive questioning, and secondary victimisation. Privacy was often compromised in shared hospital spaces. These issues highlight the need for standardised trauma‐sensitive training and reflect broader gender norms and victim‐blaming attitudes that increase survivor distress.

Instead of asking how I was coping, they wanted to know what I wore, what time I left home, and who I spoke to. I felt blamed for the violence I suffered.

(Survivor, 2)

Confidentiality is almost difficult here. The space is small, people are always coming in and out, and survivors can't trust that their stories will stay private.

(OCC staff, Dhaka)

### 3.4 Theme 4: Expectations of Continuity

Insufficient coordination among agencies supporting survivors was a significant barrier. Although OCCs are mandated to integrate medical, legal, and psychosocial services, staff reported fragmented systems, limited inter‐agency communication, and reliance on manual follow‐up. This lack of continuity often forced survivors to navigate multiple bureaucracies on their own, with no assurance that referrals to legal or social support were completed. Many survivors reported feeling abandoned after the initial crisis response, which reduced trust in the system.

After my first treatment, nobody contacted me. They said I could get legal help, but I never heard from anyone again. I had to find my own way, which was confusing and scary. It felt like the support ended as soon as I walked out of the room.

(Survivor, 20)

There is no shared system for tracking survivor cases. We don't always know if she received legal help after medical care. We depend on manual follow‐up, and sometimes we lose touch.

(OCC staff, Dhaka)

### 3.5 Theme 5: Absence of Feedback and Oversight Mechanisms

Bangladesh has a comprehensive policy framework for addressing GBV, but implementation at the facility level is inconsistent. As a result, survivors rarely have opportunities to provide feedback, and staff report limited accountability mechanisms. To address these challenges, OCCs should include survivor perspectives in monitoring and evaluation processes to ensure programs are effective and not merely symbolic.

The government talks about protecting women, but when you come here, you see how little has changed. Promises are made on paper, but the rooms are crowded, staff are missing, and survivors are not even asked how the service worked for them.

(Survivor, 21)

Policies are strong, but the local implementation is weak. We lack mechanisms to evaluate the quality of services or gather feedback from survivors. Without monitoring, centres risk becoming symbolic rather than transformative.

(OCC staff, Cox's Bazar)

## 4 Discussion

This study explored the experiences of survivors and frontline providers with hospital‐based OCC in Bangladesh, identifying gaps between expectations of survivor‐centred care and actual service delivery. The findings demonstrate ‘responsiveness' encompasses both individual provider behaviour and institutional design, including service entry processes, privacy protection, sector coordination, and the availability of meaningful channels for survivor voice and accountability. Survivors sought OCC services with expectations of immediate safety, confidentiality, respectful treatment, and coordinated support; however, these expectations were not consistently fulfilled. Limited awareness of OCCs before crisis events indicates that access is largely contingent on chance encounters and intermediaries, rather than on proactive, rights‐based pathways [31]. From a health system responsiveness perspective, access encompasses not only service availability but also whether individuals can reach services in a timely and informed manner [32]. In practice, survivors frequently accessed OCCs through police or emergency referrals, which positions formal institutions as gatekeepers.

A key finding is that survivors' awareness of OCCs is typically limited before experiencing a crisis. Most survivors learned about OCC services only after being referred by police officers or hospital staff, suggesting minimal community‐level knowledge of these centres. Previous research on GBV services in South Asia also identifies uneven information dissemination, resulting in the underrepresentation of vulnerable populations among service users [33, 34, 35]. This reliance on law enforcement and medical institutions as primary entry points may restrict access for survivors who are hesitant to involve the police or who do not utilise formal health services [36]. This referral pathway can undermine prompt attention and survivor autonomy, especially for individuals who fear stigma, retaliation, or adverse interactions with law enforcement. This pattern aligns with evidence from other LMICs, where institutional pathways, rather than self‐referral, frequently determine access to integrated services [36]. From a health system responsiveness perspective, reliance on intermediaries may undermine survivor autonomy and delay timely access to support.

Survivors in rural or semi‐urban areas encounter additional obstacles, such as extended travel times and limited transportation options, which underscore accessibility challenges. Staff consistently identified shortages in personnel and limited institutional capacity as significant barriers to timely and comprehensive care [37]. Delays in accessing counselling services, especially during evenings, holidays, or emergencies, were reported at both study sites. Staff attributed these delays to insufficient personnel and high caseloads, rather than individual neglect or unwillingness, reflecting broader challenges in public‐sector service delivery in LMICs, where resource constraints impede the provision of continuous, trauma‐informed support [38]. Implementing community‐facing information strategies and establishing multiple entry points beyond police referral are likely central to enhancing responsiveness in GBV services.

Survivor accounts further illustrate that resource constraints and workload pressures contribute to inconsistent service experiences. Within the GRPS framework, these limitations reduce both the availability and quality of services, particularly for survivors requiring immediate psychosocial assistance [37]. When staffing and space are limited, it becomes challenging to provide timely counselling, clear explanations, and adequate follow‐up, which contributes to perceptions of rushed or fragmented care [37]. These findings align with the concept of ‘stress proliferation’ within systems, wherein institutional shortages can lead to diminished quality, weaker coordination, and reduced survivor trust [11, 25]. Survivors reported varied experiences regarding service sensitivity at OCCs. Some described empathetic interactions, while others perceived questioning as judgmental or intrusive.

Privacy and confidentiality are identified as essential components of acceptable and dignified care. Shared hospital spaces and frequent interruptions compromise confidentiality, which is particularly consequential in GBV contexts where disclosure entails significant social and safety risks K [2]. Physical infrastructure and institutional routines significantly shape survivors' perceptions of care. Within the GRPS framework, these experiences relate to acceptability, underscoring the importance of dignity, respect, and privacy in service delivery [39]. The variability in survivor experiences suggests that both individual staff behaviour and broader organisational factors, such as space limitations and workload pressures, influence service sensitivity. The lack of trauma‐informed, culturally sensitive care and robust accountability mechanisms risks perpetuating the same harmful norms these services are intended to address [40, 41]. Enhancing physical privacy, establishing clear confidentiality practices, and adopting trauma‐informed communication are potentially low‐cost yet high‐impact strategies for improving perceived dignity and trust.

Across survivor narratives, gaps in coordination between medical care, psychosocial support, and legal or social referrals consistently disrupted continuity of care, reflecting a broader challenge in LMIC health systems, where siloed services hinder cross‐sectoral collaboration [41]. Despite OCCs' policy mandate to integrate health, legal, and psychosocial services, the absence of systematic referral and follow‐up mechanisms undermines continuity of care [13]. Survivors expressed uncertainty regarding follow‐up after initial medical treatment, and staff noted the absence of shared case‐tracking systems and reliance on manual processes. This fragmentation often required survivors to navigate legal or social services independently, which many found confusing or distressing [42]. Health system responsiveness is defined as the extent to which services fulfil patients' expectations regarding dignity, prompt attention, confidentiality, and autonomy [43]. These expectations significantly influence perceptions of service quality and trust in health systems [44]. From a responsiveness perspective, this issue is not merely technical; it directly influences whether survivors perceive care as coherent, timely, and supportive beyond the immediate crisis.

The current study findings indicate that current access pathways may undermine survivors' expectations of agency and early intervention. Experiences of care within OCCs might be influenced by survivors' expectations and actual service delivery [36]. Survivors reported inconsistent sensitivity, privacy concerns, and resource limitations that influenced their perceptions of service quality. These factors are particularly significant in the context of GBV, where trust and dignity are essential for recovery and continued engagement with services [45]. Fragmented coordination among medical, legal, and social components disrupted continuity of care, challenging survivors' expectations for integrated support [13]. A key finding of this study is the absence of formal mechanisms for survivor feedback and service monitoring at the facility level.

Survivors reported no opportunities to evaluate their experiences or contribute to service improvement, and staff similarly described limited accountability structures. Limited inter‐agency coordination exemplifies the institutional siloing that characterises many public sector responses to complex social issues [2, 41]. Without legally binding protocols, cross‐sectoral collaboration remains ad hoc and relies on individual initiative rather than systemic design. Implementing survivor‐tracking systems and inter‐agency referral protocols would constitute significant progress toward holistic care [46, 47]. A persistent challenge in policy design and monitoring is the frequent exclusion of survivors' perspectives. When survivor input is absent from service evaluation, initiatives often become symbolic rather than substantive [41, 48]. The near absence of routine survivor feedback mechanisms indicates a significant accountability deficit. In the absence of confidential and safe channels for expressing concerns, survivors' expectations cannot meaningfully inform service improvement. Enhancing survivor feedback mechanisms supports broader initiatives to amplify patient voice and promote accountability within health systems [6]. Incorporating survivor perspectives through confidential feedback mechanisms, periodic independent monitoring, and survivor‐informed advisory input aligns with both GRPS accountability principles.

## 5 Conclusion

The findings demonstrate a gap between survivors' expectations for timely, dignified, and coordinated care and the actual delivery of OCC services. Enhancing health system responsiveness through improved access pathways, survivor‐centred accountability mechanisms, and strengthened coordination is essential to ensure that OCCs address the expectations and needs of GBV survivors in Bangladesh.

### 5.1 Limitations

This study has several limitations. Data collection was limited to two OCCs, which may not reflect experiences in other regions of Bangladesh. Recruitment was facilitated by OCC staff following crisis care, which may have influenced participation and introduced perceived pressure, despite assurances of voluntariness. This context may also have contributed to social desirability bias in participants' accounts of services. Certain survivor groups, such as transgender individuals and persons with disabilities, were not represented, thereby limiting insight into intersectional barriers. Transcripts were not returned to participants due to safety and confidentiality concerns in the hospital‐based GBV setting, which may have limited opportunities for participant validation. The sensitive nature of the topic may have affected participants' willingness to disclose information. Additionally, translation from Bengali to English may have resulted in minor loss of nuance. These limitations should be considered when interpreting the study's findings.

### 5.2 Implications

The results of this study have important implications for improving the responsiveness of health systems to survivors of gender‐based violence. Enhancing access to OCC requires proactive communication at the community level, allowing survivors to independently identify available services instead of depending exclusively on police or emergency referrals. Greater awareness aligns with survivors' expectations for timely and autonomous access to care.

The quality and acceptability of care in OCC rely on ongoing attention to privacy, dignity, and sensitivity during service delivery. Health systems should prioritise continuous training and institutional support for frontline providers to ensure that survivor‐centred principles are embedded in routine practice, particularly in resource‐limited settings.

Fragmented coordination among medical, legal, and social services diminishes survivors' expectations for continuity of care. Strengthening referral pathways and intersectoral communication is necessary to ensure that integrated service models function effectively from the survivor's perspective.

The lack of formal mechanisms for survivor feedback constitutes a major accountability gap. Health systems should establish participatory mechanisms, such as confidential feedback channels, survivor advisory input, and community‐based monitoring, to enable survivors to express expectations, report concerns, and contribute to service improvement.

Incorporating survivor perspectives into governance structures is crucial for building trust and ensuring that integrated gender‐based violence services remain responsive to the needs and expectations of intended beneficiaries.

## Author Contributions

**Mst Sabrina Moonajilin:** conceptualisation, methodology, writing – original draft. **Sayema Mubashshira:** conceptualisation, methodology, data collection, writing – review and editing. **Md Khalid Ibne Kamal:** data curation, writing – review and editing. **Rajib Ul Islam:** data curation, writing – review and editing. **Labina Taher:** data curation, writing – review and editing. **Tabeen Taher:** data curation, writing – review and editing. **Sabkat Kamal:** conceptualisation, data curation, writing – review and editing.

## 1 Funding

The authors have nothing to report.

## Ethics Statement

The ethical aspects of this study were also reviewed and approved by the Biosafety, Biosecurity, and Ethical Review Board of Jahangirnagar University, Savar, Dhaka‐1342, Bangladesh [Ref No: BBEC, JU/M 2022/21 (6)]. All responses were anonymous, ensuring data confidentiality. All participants provided their informed consent to participate in the study after being informed about the purpose of the study.

## Conflicts of Interest

The authors declare no conflicts of interest.

## Supporting information

Consolidated criteria for reporting qualitative studies (COREQ): 32‐item checklist.

Interview guidelines.

## Data Availability

The data can be obtained from the corresponding author upon request.
