# Supplementary material for: Survivor Expectations and Experiences of One‐Stop Crisis Centres in Bangladesh: A Qualitative Study of Health System Responsiveness to Gender‐Based Violence
Source: Health Expect. 2026 Mar 27;29(2):e70643. doi: 10.1111/hex.70643 (PMC13125724; doi:10.1111/hex.70643)
Supplement: Supplementary file 1 — Consolidated criteria for reporting qualitative studies (COREQ): 32‐item checklist. [file HEX-29-e70643-s001.docx]

**Consolidated criteria for reporting qualitative studies (COREQ): 32-item checklist**

| No | Item | Guide questions/description | Answer |
| --- | --- | --- | --- |
| **Domain 1: Research team and reflexivity** | | | |
| Personal Characteristics | | | |
| 1 | Interview/facilitator | Which author/s conducted the interview or focus group? | The second author conducted all the interviews. The fifth and sixth authors helped her. |
| 2 | Credentials | What were the researcher's credentials? E.g. PhD, MD | First author: MPH  Second (MPH)  Third (MPH)  Fourth (MS)  Fifth (Student)  Sixth (Student)  Last (PhD) |
| 3 | Occupation | What was their occupation at the time of the study? | as mentioned in question 2. |
| 4 | Gender | Was the researcher male or female? | All authors are female except the fourth. Not specified in the report. |
| 5 | Experience and training | What experience or training did the researcher have? | The researcher had prior experience in conducting interviews. Not specified in the report. |
|  | Relationships with participants | | |
| 6 | Relationship established | Was a relationship established before the study commencement? | There is no relationship between researchers and participants (specified in the main report). |
| 7 | Participant knowledge of the interviewer | What did the participants know about the researcher? e.g. personal goals, reasons for doing the research | A participant information sheet that explained the research in detail was provided before conducting the interviews. |
| 8 | Interviewer characteristics | What characteristics were reported about the interviewer/facilitator? e.g. Bias, assumptions, reasons, and interests in the research topic | The interest in the research topic was explained in the introduction of the participant information sheet. Part of that explanation is specified in the introduction section of this report. |

|  | **Domain 2: study design** | | | |
| --- | --- | --- | --- | --- |
| No | Item | | Guide questions/description | Answer |
| Theoretical framework | | | | |
| 9 | Methodological Orientation and Theory | | What methodological orientation was stated to underpin the study? e.g. grounded theory, discourse analysis, ethnography, phenomenology, content analysis | A content analysis. It is explained in the report. |
| Participant selection | | | | |
| 10 | Sampling | | How were participants selected? e.g. purposive, convenience, consecutive, snowball | Convenience. It is explained in the text. |
| 11 | Method of approach | | How were participants approached? e.g. face-to-face, telephone, mail, email | Face-to-face. It is explained in the text. |
| 12 | Sample size | | How many participants were in the study? | 36 participants. Specified in the text. |
| 13 | Non-participation | | How many people refused to participate or dropped out? Reasons? | None of the participants dropped out. |
|  | Setting | | | |
| 14 | The setting of data collection | | Where was the data collected? e.g. home, clinic, workplace | Data was collected face-to-face (as specified in the report). Specified in the text. |
| 15 | Presence of non-participants | | Was anyone else present besides the participants and researchers? | No one else was present at the time of the interview. |
| 16 | Description of sample | | What are the important characteristics of the sample? e.g. demographic data, date | survivors and service providers regarding OCC services in Bangladesh |
|  | Data collection | |  |  |
| No | Item | | Guide questions/description | Answer |
| 17 | Interview guide | | Were questions, prompts, and guides provided by the authors? Was it pilot-tested? | The questions were created by the principal author and revised by the research team. The interview was practice-tested with two participants.  The research group members provided feedback regarding the type and tone of the questions. |
| 18 | Repeat interviews | | Were repeat interviews carried out? If yes, how many? | No. All the interviews were carried out once. |
| 19 | Audio/visual recording | | Did the research use audio or visual recording to collect the data? | The interviews were audio-recorded. It is explained in the report. |
| 20 | Field notes | | Were field notes made during and/or after the interview or focus group? | Field notes were made. |
| 21 | Duration | | What was the duration of the interviews or focus groups? | Between 35 and 55 minutes. Explained in the text. |
| 22 | Data saturation | | Was data saturation discussed? | Data saturation was used. It is specified in the report. |
| 23 | Transcripts returned | | Were transcripts returned to participants for comment and/or correction? | We gave the option to return the results to the interviewee as per request. None of the participants requested the result. |
|  | **Domain 3: analysis and findings** | | | |
|  | Data analysis | | | |
| No | Item | Guide questions/description | | Answer |
| 24 | Number of data coders | How many data coders coded the data? | | The main researcher coded all the data. The rest of the authors checked the names of the domain summary themes and codes, and suggestions were provided to make changes. |
| 25 | Description of the coding tree | Did the authors describe the coding tree? | | A coding tree was followed. |
| 26 | Derivation of themes | Were themes identified in advance or derived from the data? | | The themes identified were derived from the data. This is explained in the methods. |
| 27 | Software | What software, if applicable, was used to manage the data? | | Coding was done on the printed form of the transcripts. |
| 28 | Participant checking | Did participants provide feedback on the findings? | | No. |
| Reporting | | | | |
| 29 | Quotations presented | Were participant quotations presented to illustrate the themes/findings? Was each quotation identified? e.g. participant number | | Quotations were presented, and participants were anonymised using numbers, e.g. Participant 1. |
| 30 | Data and findings are consistent | Was there consistency between the data presented and the findings? | | There is consistency between the data presented and the findings. |
| 31 | Clarity of major themes | Are major themes presented in the findings? | | Themes were presented as subheadings of the results section. |
| 32 | Clarity of minor themes | Is there a description of diverse cases or a discussion of minor themes? | | Codes were not specified in a special format since we wanted to focus on the story itself. |
