# Supplementary material for: Survivor Expectations and Experiences of One‐Stop Crisis Centres in Bangladesh: A Qualitative Study of Health System Responsiveness to Gender‐Based Violence
Source: Health Expect. 2026 Mar 27;29(2):e70643. doi: 10.1111/hex.70643 (PMC13125724; doi:10.1111/hex.70643)
Supplement: Supplementary file 2 — Interview guidelines. [file HEX-29-e70643-s002.docx]

### **Interview guidelines**

***Introductions and information about the research:***

I am a student of the Department of Public Health and Informatics at Jahangirnagar University. As part of my master’s program, I am conducting a research project titled Assessment of Service Delivery at One-Stop Crisis Centre Focusing on Gender-Based Violence in Bangladesh.

***I just want to double-check a few things before we begin today:***

- Did you read and understand the information sheet?
- Have you got satisfactory answers to all your questions?
- Have you completed the consent form?
- Are you aware that you can contact the Research Ethics Advisory Team if you have any questions or concerns about the ethical conduct of this research project?
- Are you aware that you have the option of withdrawing with no remark or penalties?
- Do you realise that this interview will be recorded?

**Demographic and rapport-building questions:**

- How was your day?
- What do you study/do?
- Where do you live(urban/rural)?

Is there anything else you'd like to ask before we start? Right now, I'm going to start recording audio.

For Survivors

Section A: Background Information

- Age
- Education level
- Marital status
- Occupation
- Family composition

1. How did you first hear about the OCC?
2. What made you decide to come here?
3. What challenges did you face in reaching the OCC (e.g., distance, cost, social stigma)?
4. Were there alternative services you considered before coming?
5. Can you describe your first experience at the OCC?
6. What types of services did you receive (medical, legal, psychosocial, shelter)?
7. How did staff treat you (respect, empathy, confidentiality)?
8. Were your privacy and dignity maintained during treatment and counselling?
9. Did you feel blamed, judged, or supported during the process?
10. Were you referred to legal aid, police, or shelters? If yes, how was the follow-up?
11. Did you feel abandoned or supported after initial treatment?
12. Were you ever asked for feedback about services?
13. What changes would make OCCs more helpful for survivors like you?
14. If you could change one thing to make OCCs more effective for survivors, what would it be?

**For Staff**

Section A: Background Information

- - Age
  - Education level
  - Marital status
  - Occupation
  - Family composition

1. In your view, how aware are community members about OCC services?
   How do survivors usually reach OCCs (referrals, self-referral, others)?
2. What barriers reduce survivor access, especially for rural, unmarried, or young women?
3. What is your role in providing OCC services?
4. How do you ensure survivor confidentiality and sensitivity?
5. What challenges do you face in delivering quality care?
6. Are there enough trained staff, especially during nights and emergencies?
7. What resources are missing (infrastructure, equipment, training, shelter facilities)?
8. How do shortages affect survivor experiences?
9. How does OCC coordinate with police, health, and legal services?
10. Are there referral tracking systems? If not, what happens after survivors leave OCCs?
11. What are the main barriers to effective coordination?
12. Are there monitoring or accountability mechanisms in place?
13. How is survivor feedback collected and used
14. What policy or program changes are needed to strengthen OCCs?
15. If you could change one thing to make OCCs more effective for survivors, what would it be?

**General Conclusion:**

Is there anything else you think we should discuss that we haven't?

(If they do, explore what they're saying. If not, or after they have finished, thank them, and conclude the interview. Turn off all recording devices and software.)
